# Advancements of algae-involved cancer treatment

**DOI:** 10.52601/bpr.2024.240055

**Published:** 2025-08-31

**Authors:** Tian Qiu, Xingrun Li, Hui Sun, Simeng Zhang, Yan An, Jianxiang Li, Xiaoyan Zhong

**Affiliations:** 1 Department of Toxicology, School of Public Health, Jiangsu Key Laboratory of Preventive and Translational Medicine for Major Chronic Non-communicable Diseases, MOE Key Laboratory of Geriatric Diseases and Immunology, Suzhou Medical College of Soochow University, Suzhou 215123, Jiangsu, China

**Keywords:** Algae, Nanomaterials, Engineering strategies, Biological effects, Cancer treatment

## Abstract

Algae, especially microalgae, are versatile in terms of nutrition, feed, biofertilizers, biofuel, and so on. In the realm of oncotherapy, algal extracts have been extensively used as anti-cancer active ingredients; however, what has been ignored is the anti-cancer value induced by themselves. Thanks to their unparalleled advantages, for example, intrinsic tumor homing, immunogenicity, and *in situ* production of anti-cancer agents, algae pave a new way in anti-cancer research. Algae have been reported about selective cytotoxicity to cancer cells and could work for oxygen-dependent strategies such as photodynamic therapy, owing to their natural photosynthetic abilities. Interestingly, integrating with customized nanomaterials (NMs), algae have been demonstrated to have unprecedented potential in overcoming barriers to existing treatment methods. Thus, in this review, starting from the classification of algae, the diverse effects of algae are thoroughly introduced, followed by the current engineering strategies of algae; lastly, the emerging development of algae-based therapeutics is timely summarized with an emphasis on the intelligent creation of biohybrid systems by choosing algae and tailored NMs. This review presents a comprehensive exploration of engineered algae-involved innovative cancer therapy, with a discussion of the future challenges and outlook, which will help design creative therapy paradigms and facilitate their clinical applications.

## INTRODUCTION

Cancer is a pervasive global health challenge that continues to impose significant burdens on societies worldwide (Xin *et al.* 2023). The overall trends in cancer incidence and mortality vary across regions, with notable declines observed in certain populations such as in the United States, while emerging economies face a growing cancer epidemic (Siegel *et al.* 2023). In China, the crude incidence and mortality rates of cancer have steadily risen, highlighting the urgency for novel treatment approaches (Sepich-Poore *et al.* 2021). Algae are the main cause of water blooms, which are one of the problems of ecological management. From a public health perspective, if environment-threaten algae can be used in cancer treatment, this “one-stone-for-two-birds strategy” is not only a novel achievement in combating cancer but also can provide help for aquatic environment treatment, making great hygienic and economic importance to turn environmentally harmful algae into potent anti-cancer drugs (Yang *et al.* 2024b). Being highly abundant in nature, readily available, and diverse, algae has recently attracted substantial attention in cancer treatment (Ren *et al.* 2021). For example, algae extracts that contain phycocyanin, fucoidan, *etc*., have been proven as effective anti-cancer active ingredients with high biological activity, low drug resistance, and small side effects for decades in the vast space of drug research and development (Al-Malki 2020; Liu *et al.* 2022a; van Weelden *et al.* 2019). In addition, anti-oxidants such as β-carotene, and vitamins C and E in the extracts can effectively scavenge free radicals and reduce the occurrence and progression potential of cancer (Abbasian *et al.* 2023; Tavares *et al.* 2023). Moreover, other algae extracts such as algal pigments or nucleic acids, can inhibit the growth and spread of cancer cells through different mechanisms, thus featuring potential therapeutic value in cancer treatment (Martínez Andrade *et al.* 2018).

Previously neglected algae with similar anti-cancer value has entered the scene recently (Shikov *et al.* 2020). Algae are highly productive with exuberant vitality; many species have been commercially used as nutritional and dietary additives, proving to be a practical and safe oral pharmaceutical formulation. Compared with other used microorganisms in cancer therapy, such as bacteria, viruses, and fungi, algae exhibit more comprehensive functions including versatility, unique photosynthetic oxygen (O_2_) production capacity and motion-targeting ability, nonaggressive to cells or tissues, and different administration routes. In detail, the unique surface structure, and locomotion characteristics such as phototaxis, chemotaxis, magnetotactic, and degradability provide strong support for drug delivery, which can improve the targeting and stability of drugs (Liu *et al.* 2022b). In addition, algae are rich in photosynthetic pigments, which are sensitive to light and thus can be used as both O_2_ generators or even photosensitizers (PSs), for fluorescence and photoacoustic imaging-guided O_2_-dependent treatments like radiotherapy (RT) and photodynamic therapy (PDT), respectively (Cen *et al.* 2021; Qiao *et al.* 2020). Therefore, the application of algae can realize the “integration of diagnosis and treatment” in the field of oncology.

**Figure 1:**
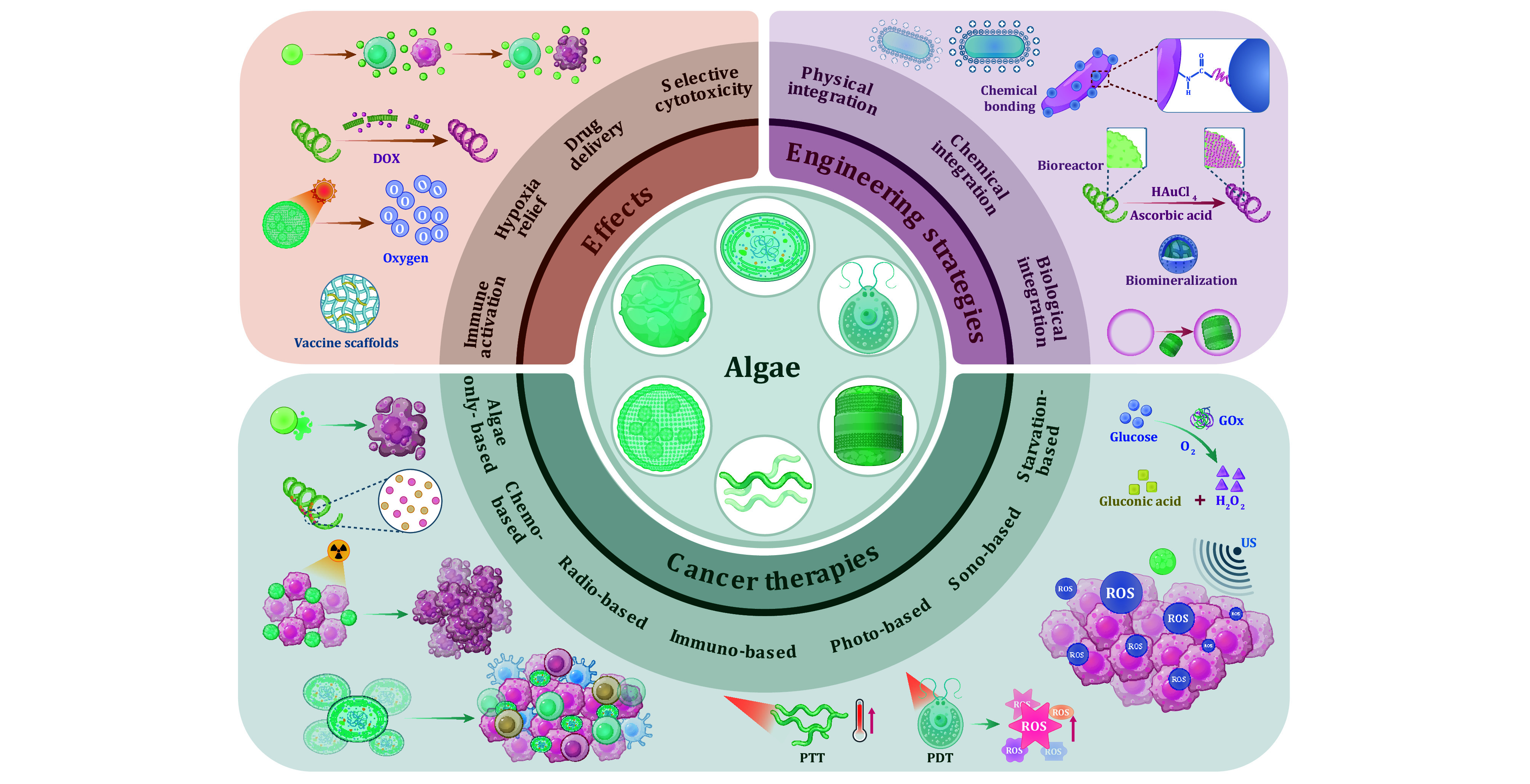
The exploration of algae-involved platforms in cancer therapy

In this paper, explorations into algae against cancer were timely and comprehensively reviewed. Firstly, beginning with the classification of algae that is mainly divided into prokaryotic and eukaryotic algae, the biological characteristics that are necessary conditions for anti-cancer were clearly described. Secondly, the biological effects and applications of algae in cancer therapy were well-documented, including their cytotoxicity, drug delivery, hypoxia relief, and immune activation, *etc*. Thirdly, engineering strategies that are booked for constructing engineered algae were systematically illustrated where algae can be modified through physical, chemical, and biological strategies, making them not only maintain their inherent properties but also become multifunctional to boost anti-cancer efficacy. Finally, the specific functions and roles of algae in different remedial models were described in detail (Fig. 1). This review will provide a detailed introduction to algae against cancer, aiming to comprehensively explore the possibilities of algae in cancer therapy and to provide researchers with new thoughts, thus promoting the future innovation of algae.

## THE CLASSIFICATION OF ALGAE

Algae usually refer to aquatic organisms that have chlorophyll and can be used for photosynthesis. There are various classification methods for algae. Algae can be divided into *Cyanobacteria* (*Cyano*), green algae, red algae, *etc*. according to different algae pigments (Funk *et al.* 2017). Regarding the different sizes and morphology of algae, they can also be divided into large algae composed of multiple cells and microalgae composed of single cells (Qian *et al.* 2020). Among them, microalgae have received widespread attention due to their anti-oxidant, anti-bacterial, anti-viral, anti-tumor, and other effects (Abd El-Hack *et al.* 2019; Frazzini *et al.* 2022; Martínez Andrade *et al.* 2018). Compared with large seaweed, microalgae have more potential in biomedical applications due to their advantages of fast growth, easy modification and high biocompatibility (Khavari *et al.* 2021). From another aspect, algae can also be divided into prokaryotic and eukaryotic subtypes according to the different intracellular organelles (Table 1) (Basheva *et al.* 2018; Gélabert *et al.* 2018; Generalić Mekinić *et al.* 2023; Kamble *et al.* 2018; Li *et al.* 2022, 2024b; Lin and Pakrasi 2019; Mehdizadeh Allaf and Peerhossaini 2022; Yakoub *et al.* 2024; Yang *et al.* 2023a; Zhao *et al.* 2019; Zhou *et al.* 2016).

**Table 1:** Physicochemical properties of algae

Algae	Size (μm)	Zeta (mV)	Functional group	Bioactive components
Prokaryotic algae				
*Cyano*	1–100	~(−6) – ~ (−30)	-COOH	Phenolic acids (2.97–5.87 mg/g)
			PO_4_^3-^	Phycocyanin (0.78–4.06 mg/g)
			-NH_2_	Flavonoids
			-CH_2_/CH_3_	Terpenoids
			-C=O	Tannins
			-CHO	Peptides
*SP*	Length: 100–500	~(−20) – ~(−40)	-OH	Phycocyanin (100–300 mg/g)
	Width: 5–10		-C=O	Polysaccharide of spirulina platensis
			-CH_2_/CH_3_	(50 mg/g)
				Carotenoid (3.5±0.2 mg/g)
				Proteolytic peptides
				Polyphenol
Eukaryotic algae				
*Chl*.	2–10	~0 – ~(−30)	-COOH	Polysaccharides (100–200 mg/g)
			-OH	Chlorella growth facter (40 mg/g)
			-NH_2_	Carotenoid (0.22–3.20 mg/g)
			-CH_2_/CH_3_	Glycoprotein
			-C=O	
*C*. *reinhardtii*	5–10	~(−10) – ~(−30)	-COOH	Polysaccharides (197 mg/g)
			-OH	Glycoproteins
			-NH_2_	Carotenoid
			-C=O, -P=O	
Diatom	Length: 6–15	~(−15) – ~(−40)	-NH_2_	Polysaccharides (50–240 mg/g)
	Diameter: 1–2		-Si-OH	Fucoxanthin (2.2–21.7 mg/g)
			-COOH	Flavonoids

### Prokaryotic algae

Prokaryotic algae, mainly referred to *Cyanophyta*, usually have a simple structure. Although without chloroplasts, the photosynthesis of oxygenation can also be carried out through chlorophyll (Dismukes *et al.* 2001; Gao *et al.* 2016). There are many photosynthetic membranes, also called cystoids, in the cytoplasm of *Cyano*, and many photosynthetic pigments such as chlorophyll a and carotenoids are attached to them where O_2_ is produced via photosynthesis. *Cyano* also has high biocompatibility, which was further validated in promoting wound healing and alleviating myocardial ischemia (Cohen *et al.* 2017; Hopfner *et al.* 2014). Based on the photosynthesis and biocompatibility of *Cyano*, the produced O_2_ via photosynthesis can be used to relieve the hypoxic microenvironment of tumors, benefiting O_2_-dependent treatment modalities (Huo *et al.* 2020b; Liu *et al.*^ 2020^; Qi *et al.* 2021; Sun *et al.* 2020). Belonging to an edible *Cyanophyta*, *Spirulina* (*SP*) is named for its loose or tight-packed regular spiral curvature, with 100–500 μm in length and 5–10 μm in width. It is abundant with a variety of nutrients, exhibiting anti-oxidative, anti-inflammatory, anti-viral, and anti-tumor performances (Asghari *et al.* 2016; Belay *et al.* 1993; Finamore *et al.* 2017). Since 2003, *SP* has been certiﬁed as safe at the population level and it worked as an outstanding drug delivery system (DDS) due to not only its large, negatively-charged surface which is easy for functional modification and drug loading but also the flexible movement and rotation ability to target lungs and intestines (An *et al.* 2024; Wang *et al.* 2019; Yan *et al.* 2019; Zhong *et al.* 2021c). Moreover, the intracellular fluorescent chlorophyll can also be used for *in vivo* imaging monitoring (Yan *et al.* 2017; Zhong *et al.* 2020a). Just as important is its degradability to guarantee excellent biocompatibility and biosafety (Ciferri 1983).

### Eukaryotic algae

Unlike prokaryotic algae, eukaryotic algae, which are mainly divided into ten phyla such as Chlorophyta, and Diatoma, *et al*, perform photosynthesis in chloroplasts (Rockwella *et al.* 2015). Among them, green algae is the most widely distributed, and it is also one of the most important raw materials for consumption and production (Georgianna and Mayfield 2012; Haq *et al.* 2019). *Chlorella* (*Chl.*) is a representative example that can be widely found in nature, being rich in protein, chlorophyll, fiber, vitamins, and minerals. *Chl.* has been proven effective in anti-oxidants, strengthening the immune system, and ameliorating cholesterol and blood pressure (Bito *et al.* 2020; Chou *et al.* 2012; Yang *et al.* 2016). Importantly, *Chl.* can use photosynthetic pigments in chloroplasts for photosynthesis, which is more efficient than that of prokaryotic algae (Mauzerall 1972; Price *et al.* 2012). Based on this, a large number of reactive oxygen species (ROS) can be produced by *Chl.* under light irradiation, and so did *Chlamydomonas reinhardtii* (*C. reinhardtii*) (Napolitano *et al.* 2020; Wang *et al.* 2022; Zhou *et al.* 2019). Over than that, *C. reinhardtii* can sense and tend to the visible light source at an extremely fast speed, about 100–200 μm/s (Harris 2001; Yasa *et al.* 2018). This phototaxis endows *C. reinhardtii* with the possibility of preparing a biohybrid algae microswimmer system as a cargo delivery platform (Akolpoglu *et al.* 2020).

Diatoms are a kind of single-cell plants coated with silicon cell walls, and most are silicon dioxide that is connected by several or many individual cells into a variety of groups (Losic *et al.* 2009; Townley *et al.* 2008). Natural silica is a biocompatible, thermally and chemically stable material, providing additional adsorption points for other drugs or nanoformulations to facilitate drug delivery (Aw *et al.* 2011; Losic *et al.* 2010; Maher *et al.* 2016).

Apart from these, brown algae is a kind of large algae, which has the characteristics of fast growth, large yield, and high economic value (Carroll *et al.* 2021). Brown algae are rich in polysaccharides, polyphenols, sterols, terpenoids, proteins, and other active ingredients, which have anti-cancer, anti-bacterial, anti-inflammatory, anti-viral, blood pressure lowering, and diabetes treatment activities that exhibit an important position in food, drugs and health products and other fields (Couteau and Coiffard 2020; López-Hortas *et al.* 2021; Senthilkumar and Kim 2014). In detail, the fucoidan in brown algae plays a direct anti-cancer role by inhibiting the cell cycle and promoting apoptosis; this substance can also activate natural killer cells and macrophages for subsequent immunity (Jin *et al.* 2021; Senthilkumar and Kim 2014). Interestingly, the synthesis of metal nanoparticles (NPs) with anti-cancer activity by brown algae in different ways has great potential against cancer (González-Ballesteros *et al.* 2017; Hamouda and Aljohani 2024).

## THE BIOLOGICAL EFFECTS AND APPLICATIONS OF ALGAE

With a comprehensive understanding of the structure and function of algae, more and more abundant effects of algae on cancer therapy have been discovered, mainly including selective cytotoxicity, drug delivery, hypoxia relief, as well as immune activation.

### Selective cytotoxicity

Overall, there are about 3.5% of marine plants, including algae, possess anti-cancer or cytotoxic properties according to the National Cancer Institute (Xin *et al.* 2023). For example, *Chl.* could induce a decrease in the viability of A549 (a non-small-cell lung cancer cell line) and CL1–5 (a lung adenocarcinoma cell line) via mitochondria-mediated apoptosis by down-regulating B cell lymphoma 2 (Bcl-2) (Bcl-2), X-linked inhibitor of apoptosis (XIAP), and surviving (Lin *et al.* 2017). Different from the induction of apoptosis mentioned above, the navigable robotic device called biohybrid magnetic robots (BMRs) showed cytotoxicity on cancer cells of SiHa (a cervical cancer cell line) and HepG2 (a hepatoma cell line) other than normal cell line of 3T3 (a murine fibroblast cell line); this selective toxicity might be attributed to the bioactive phycocyanin in *SP* (Yan *et al.* 2017).

Interestingly, besides the selective cytotoxicity towards cancer cells, new anti-cancer substances can be *in situ* produced within tumor cells by algae-based biomaterials. By biosynthesis of gold nanoparticles (Au NPs) on the surface of *Shewanella algae K3259* (*S. algae*), a hybrid biosystem of Bac@Au produced anti-neoplastic tetrodotoxin (TTX), an apoptosis inducer by increasing the level of oxidative stress, which showed a potent anti-cancer ability (El-Dayem *et al.*^2013^; Wang *et al.* 2021b). Some algae also feature immunomodulatory and anti-viral properties, which facilitate the combination therapy to improve the efficacy of cancer treatment (El-Sheekh *et al.* 2022).

### Drug delivery

Some algae with special structures can be used as DDS to load chemotherapeutic or genetic drugs to target tumors when driven by external light radiation or magnetic fields (Gong *et al.* 2022; Maher *et al.* 2016; Zhong *et al.* 2020b). Doxorubicin (DOX) is the first-line drug against cancer via deoxyribonucleic acid (DNA) damage, ROS production, apoptosis, autophagy, and other mechanisms (Hu *et al.* 2023; Kciuk *et al.* 2023; Li *et al.* 2018; Zhong *et al.* 2023). However, this prolonged use of DOX can result in severe drug resistance and non-specific toxicity to healthy cells (Cagel *et al.* 2017). With the burgeoning of nanomedicine, it is an effective method to transport therapeutic agents to specific sites using DDS (Imran *et al.* 2024; Lin *et al.* 2023). The special structure, surface activity, and targeted transport of algae make them a good candidate for drug delivery (Liu *et al.* 2022b).

By electrostatic interaction, algae with a negatively charged surface can deliver oppositely charged DOX to enhance chemotherapy. For example, the DDS of SP@DOX was successfully constructed with high drug loading efficiency, high targeting efficiency, and good biological safety (Zhong *et al.* 2020b). In another study, diatom-oriented porous silicon nanoparticles (Si NPs) by mechanical crushing and magnesiothermic reduction also showed improved drug loading efficiency owing to the high surface area and tunable pore morphology (Maher *et al.* 2016). Similarly, DOX-loaded biohybrid microrobot multimers (BMMs), and genetically engineered diatom *Thalassiosira pseudonana* with the payload of camptothecin (CPT) and its derivative 7-ethyl-10-hydroxy-CPT (SN38), also exhibited high drug loading efficiency, pH-triggered drug release, along with low side effect (Delalat *et al.* 2015; Gong *et al.* 2022).

### Hypoxia relief

Considering the insufficient O_2_ content in the tumor microenvironment (TME), hypoxia relief strategies can improve the outcome of treatment that depends largely on O_2_. As an organism that produces O_2_ through photosynthesis, algae can be used to relieve the hypoxic TME for tumor inhibition. For this purpose, an autotrophic light-triggered green affording-oxygen engine (ALGAE) biological system composed of *Chl.* and calcium alginate was designed. Under the protection of alginate that avoids phagocytosis, *Chl.* Continuously produced O_2_ through decomposition of water and energy conversion, alleviated tumor hypoxia, lowered vascular endothelial growth factor (VEGF) and hypoxia-inducing factor (HIF-1α), and thus inhibited growth and metastasis of tumor cells (Zhou *et al.* 2019). In another study, *Chl.* was protected by a red blood cell membrane, and O_2_ was effectively produced *in situ* under light irradiation, thus increasing tissue oxygenation (Qiao *et al.* 2020).

### Immune activation

Vaccination is commonly used to prevent infectious diseases, however, due to the ability of vaccines to enhance antigen-specific immune responses, researchers have focused their attention on developing vaccines for chronic non-communicable diseases (Fan *et al.* 2023; Lin *et al.* 2022). Tumor vaccines can stimulate or strengthen anti-tumor immunity through the expression of specific, immunogenic tumor antigens, such as peptides, deoxyribonucleic acid (DNA), and ribonucleic acid (RNA), with the assistance of adjuvants, which in turn kill and remove cancer cells (Fan *et al.*^2023^; Saxena *et al.* 2021). Limited by both the low immunogenicity of tumor antigens and the immunosuppressive TME, the use of adjuvants and vaccine carriers is an effective way to improve the efficacy of immunotherapy (Kim *et al.* 2015). It has been shown that *SP* could activate the human innate immune system to inhibit tumor progression (Ge *et al.* 2019; Hirahashi *et al.* 2002). For example, the helical structure of *SP* was utilized to act as an *in situ* vaccine scaffold to enhance local hyperemia and activate anti-cancer immune responses (Qi *et al.* 2024). This type of immune adjuvant using natural materials is important for the development of cancer immunotherapy.

## ENGINEERING STRATEGIES OF ALGAE

While algae perform a wide variety of functions described above, they frequently require physical or chemical modification to enhance their characteristics or biological activity during cancer treatment. The strategies used to fabricate engineered algae mainly include physical, chemical, and biological integrations (Table 2).

**Table 2:** Engineering strategies for algae

Engineering strategies	Interaction mode	Algae	Engineered algae	Reference
Physical integration	Electrostatic adsorption	*Cyano*	ceCyan	Huo *et al.* 2020b
	Electrostatic adsorption	*Cyano*	UR-Cyan	Huo *et al.* 2021
	Electrostatic adsorption	*C. reinhardtii*	biohybrid C. reinhardtii microswimmers	Akolpoglu *et al.* 2020
	Electrostatic adsorption	*Chl.*	BMMs	Gong *et al.* 2022
	Electrostatic adsorption	*SP*	MSP hybrid microswimmers	Zhong *et al.* 2020a
	Electrostatic adsorption	*SP*	ADU@Fe-SP	Zhang *et al.* 2023
	Ionic cross-linking	*SP*	SP-Au	Hua *et al.* 2024
	Ionic cross-linking	*Chl.*	ALGAE	Zhou *et al.* 2019
Chemical integration	Chemical bonding	*Cyano*	S/HSA/ICG	Liu *et al.* 2020
	Chemical bonding	*Chl.*	CurNPs-C	Zhang *et al.* 2024b
	Chemical bonding	*Cyano*	Cyan@BPNSs	Qi *et al.* 2021
	Bioreactor	*S.algae*	Bac@Au	Wang *et al.* 2021b
	Bioreactor	*SP*	(Pd@Au)/Fe_3_O_4_@Sp.-DOX	Wang *et al.* 2019
	Bioreactor	*SP*	Au-SP@CF	Hosseini *et al.* 2022
	Biomineralization	*Chl.*	CV@CaP	Zhong *et al.* 2021b
	Biomineralization	*Chl.*	Algae@SiO_2_	Li *et al.* 2020
Biological integration	Membrane coating	*Chl.*	RBCM-Algae	Qiao *et al.* 2020
	Membrane coating	*Chl.*	M-Chl	Gao *et al.* 2022
	Membrane coating	*Chl.*	MChl-CQ-HP-NP	Gao *et al.* 2023
	Antibody binding	*T. pseudonana*	Diatom biosilica	Delalat *et al.* 2015

### Physical integration

Usually, the surface of algae is negatively charged, so positively charged materials can be physically deposited on their surface by electrostatic adsorption. For example, the phosphoric acid group on the cell membrane of *Cyano* is negatively charged, while the PEGylated photosensitizer of chlorin e6 (Ce6) is positively charged owing to the existing amide group, therefore, the complex of ceCyan containing *Cyano* and Ce6 could be produced after co-incubation. Similarly, *Cyano* loading another photosensitizer of protoporphyrin could also be prepared (Huo *et al.* 2020b). In addition to directly modifying algae with only positively charged materials, positive coating-covered algae can be further modified with negatively charged materials. For instance, up-conversion nanoparticles (UCNPs) were coated with the photosensitizer of rose bengal (RB) to form URNPs via electrostatic hybridization (Huo *et al.* 2021). Similarly, the positively charged chitosan complex deposited on the cell wall of *C. reinhardtii* could make the adhesion of NPs tighter but not affect the phototaxis and motility of biohybrid microalgae (Akolpoglu *et al.* 2020).

Magnetic micro/nano-robots are getting a lot of attention since anti-cancer drugs can be concentrated within the tumors driven by magnetic targeting. Biocompatible algae provide a natural raw material for the preparation of magnetic micro/nanorobots. For example, the positively charged precursors for superparamagnetic Fe_3_O_4_ NPs could deposited on the surface of algae. As the reaction time increased, more and more Fe_3_O_4_ NPs grew up and firmly bound to the prefabricated magnetite coating and gradually enhanced its magnetism. The prepared engineered algae displayed great potential in oncotherapy in the external magnetic field (Gong *et al.* 2022; Zhang *et al.* 2023; Zhong *et al.* 2020a). Differently, prefabricated Fe_3_O_4_ NPs could also be directly deposited on the surface of *SP* to prepare magnetic *Spirulina platensis* (MSP) hybrid microswimmers, which were able to target the tumor site driven by the magnetic field (Zhong *et al.* 2020a). Moreover, Fe_3_O_4_ NPs could also be delivered by *Chl.* through a process of dynamic magnetic self-assembly to prepare biohybrid microrobots, followed by being reversibly disassembled to achieve intelligent control (Gong *et al.* 2022).

The electrostatic adsorption-based binding method has some instability, whereas ionic cross-linking in the form of ionic bonding can form more stable hybrid complexes. For example, by co-incubating *SP* with a solution of calcium chloride and carboxymethyl chitosan-coated gold nanoclusters (Au NCs), the Au NCs could be tightly bound to *SP* by Ca^2+^ cross-linking (Hua *et al.* 2024). Similarly, C*hl.* and alginate via Ca^2+^ crosslinking were also reported (Zhou *et al.* 2019).

Electrostatic adsorption is relatively convenient and occupies the main position in the physical modification strategy, but still faces potentially unstable risks. Therefore, further development of better physical modification methods is essential.

### Chemical integration

Unlike physical interactions, chemical bonding involves forming a chemical bond between molecules, atoms, or ions. Due to the presence of peptidoglycan, polysaccharide, protein, and lipopolysaccharide (LPS), the surface of algae contains many functional groups, including amino, carboxyl, aldehyde group, hydroxyl groups, and others (Huo *et al.* 2020a). These functional groups can provide conjugated anchor points for further chemical modification of algae (Spicer *et al.* 2018). For example, indocyanine green (ICG) and human serum albumin (HSA) NPs could be combined into nanophotosensitizers of HSA/ICG via disulfide bond first. Then, the carboxyl group on their surface could be activated by 1-ethyl-3-(3-dimethylaminopropyl) carbon diimide (EDC) and N-hydroxysuccinimide (NHS), followed by amidation with the amine group on the surface of *Cyano* to form the biomimetic system of S/HSA/ICG (Liu *et al.* 2020). Similarly, curcumin (Cur) NPs with abundant carboxyl groups could also linked to amine groups of *Chl.* under the activation of EDC/NHS to prepare CurNPs-C (Zhang *et al.* 2024b). On the contrary, the carboxyl group on the surface of *Cyano* could react with the amide group of black phosphorus nanosheets (BPNSs) to form hybrid photosynthetic Cyan@BPNSs when activated by EDC/NHS (Qi *et al.* 2021).

Apart from the various functional groups for the surface engineering of algae, the special metabolic abilities afford various metabolites with the ability to transfer electrons from organic materials to metal ions, making metals easier to be reduced (Choi *et al.* 2018; Yin *et al.* 2019). Therefore, metal NPs, such as Au NPs can be *in situ* synthesized inside or outside of algae, and been used as functional materials for further applications (Park *et al.* 2016). For example, by utilizing the bidirectional electron transport function of *S. algae* (Bac), the electrons could be transferred from electron donors to terminal Au^3+^ ions, and eventually biosynthesized Bac@Au (Wang *et al.* 2021b). Differently, Au^3+^ ions could attach to *SP* via electrostatic adsorption, and further transfer to the interior of *SP* through water channels and pores of the cell membrane, where it could be reduced by the active biological compounds to successfully construct Au-SP (Hosseini *et al.* 2022). Moreover, bimetallic materials such as Pd@Au NPs could also be deposited on the *SP* to form (Pd@Au)@Sp through electroless plating, with Pd seeds as a catalytic core. Then, Fe_3_O_4_ NPs could be further deposited, forming (Pd@Au)/Fe_3_O_4_@Sp by the Sol-Gel method (Wang *et al.* 2019).

Similar to the above-mentioned methods, biomineralization installs a protective film on the algae, with simple fabrication, high biocompatibility, low cytotoxicity, and high degradation (Shen *et al.* 2004). For example, *Chl.* could be coated with a protective shell of calcium phosphate (CaP) to form CV@CaP. In detail, Ca^2+^ ions were first deposited on the surface of *Chl.* and then supplemented with phosphate ions to form CaP by *in situ* mineralization. The resulted CV@CaP had a smooth, dense surface and survived longer in the physiological environment than bare *Chl.* (Zhong *et al.* 2021b). Similarly, Algae@SiO_2_ could also be obtained by biomimetic silicification (Li *et al.* 2020).

The surface of algae has many kinds of functional groups and membrane components that provide excellent binding sites for algal modification. It is a good strategy to modify algae through chemical reactions to change their activities. However, some reagents used in the chemical reaction need to be carefully considered since they may have side effects. In subsequent studies, some mild chemical methods can be chosen to construct engineered algae.

### Biological integration

In addition to the above physicochemical strategies, algae can also be covered by cell membranes to realize the alleviated immune response in the body triggered by foreign substances, the reduced potential toxicity of the material, as well as the enhanced targeting ability (Chen *et al.* 2016; Jiang *et al.* 2017; Qiao *et al.* 2020; Xin *et al.* 2023). Typically, red blood cell membrane (RBCM) is usual as an invisibility cloak to avoid attack by the autoimmune system of the body, reducing phagocytosis of macrophages and thus more successful transport of algae to the tumor site (Qiao *et al.* 2020). Thanks to the inflammatory homing effect of the macrophage cell membrane, the coated *Chl.* (M-Chl) effectively escaped phagocytosis and actively targeted tumor tissues in response to inflammatory signals *in vivo* (Gao *et al.* 2022).

Antibody binding and bioconjugation of streptavidin-biotin via non-covalent affinity bonds have become another general strategy for microbial modification (Suh *et al.* 2019). Genetic engineering of the diatom *Thalassiosira pseudonana* allowed the improved attachment of cell-targeting antibodies by displaying the IgG binding region of protein G on the surface of biosilica (Delalat *et al.* 2015).

Briefly, algae can be physically, chemically, and biologically modified to maintain their intrinsic activity and become more multifunctional. The advantages of algae can be doubled by combining with different materials to obtain satisfactory tumor therapeutic effects. However, the main focus at present is on the surface modification of algae, and more methods are worthy of further exploration. Secondly, the combination of multiple modifications may achieve better therapeutic performance, showing a wide range of research prospects.

## CANCER THERAPY

The mainstream strategies in clinical practice have the limitation of unwanted trauma, high toxic side effects, low individual response rate, and so on; therefore, exploring novel strategies for cancer therapy is urgently needed (Liu *et al.* 2024). The aforementioned different structures and rich functions of algae inspire in design of innovative cancer treatment strategies. Up to now, algae-based treatment modalities have been expanded from traditional chemotherapy, radiotherapy, and immunotherapy, to novel photo-based, sono-based, and starvation-based therapies (Table 3). Algae can improve the outcomes of anti-cancer methods, providing efficient and safe innovative ways for the application.

**Table 3:** Algae for cancer therapy

Therapy	(Engineered) algae	Cancer	Administration route	Reference
Algae-only based	*Chl.*	Non-small-cell lung cancer	p.o.	Lin *et al.* 2017
	*C. reinhardtii*	Melanoma	i.v.	Holmes *et al.* 2022
	*C. reinhardtii*	B-cell lymphomas	p.o.	Tran *et al.* 2013
	Bac@Au	Breast cancer	i.v.	Wang *et al.* 2021b
	*SP*	/	/	Yan *et al.* 2017
Chemo-based	Diatom	Neuroblastoma, B-lymphoma	i.p.	Delalat *et al.* 2015
	Chlorella AuNRs BSA-Gel	Breast cancer	i.p.	Lee *et al.* 2019
	SpiD	Osteosarcoma	i.p.	An *et al.* 2024
	MChl	Melanoma	i.v.	Gao *et al.* 2022
	*C. reinhardtii*	Breast cancer	/	Akolpoglu *et al.* 2020
	BMMs	Cervical cancer	/	Gong *et al.* 2022
	Diatom	Breast cancer	/	Maher *et al.* 2016
	(Pd@Au)/Fe_3_O_4_@Sp.-DOX microrobots	Esophageal carcinoma renal cell carcinoma	/	Wang *et al.* 2019
	SP@DOX	Breast cancer	i.v.	Zhong *et al.* 2020b
	SP@Curcumin	Colon cancer	p.o.	Zhong *et al.* 2021c
Radio-based	SP@Curcumin	Colon cancer	p.o.	Zhong *et al.* 2021c
	SP-Au	Breast cancer, Lung cancer	i.t.	Hua *et al.* 2024
	RBCM-Algae	Breast cancer	i.t., i.v.	Qiao *et al.* 2020
	Algae@SiO_2_	Breast cancer	i.v.	Li *et al.* 2020
	CV@CaP	Breast cancer	i.v.	Zhong *et al.* 2021b
	MSP hybrid microswimmers	Breast cancer	i.v.	Zhong *et al.* 2020a
	SP@AMF	Colorectal cancer	p.o.	Zhang *et al.* 2022
Immuno-based	ADU@Fe-SP	Colorectal cancer	i.v.	Zhang *et al.* 2023
	Au@E-SP	Breast cancer	s.c.	Qi *et al.* 2024
	Chl@BP-Fe	Melanoma	i.t.	Ou *et al.* 2022
	S/HSA/ICG	Breast cancer	i.v.	Liu *et al.* 2020
	MChl-CQ-HP-NP	Melanoma	i.v.	Gao *et al.* 2023
	MChl	Melanoma	i.v.	Gao *et al.* 2022
	*Me*	Breast cancer, Colon cancer	i.t.	Yang *et al.* 2024b
	ACG gel	Breast cancer	i.t.	Zhang *et al.* 2024a
Photo-based	Au-SP@CF	Breast cancer	i.v.	Hosseini *et al.* 2022
	*Cyano*	Breast cancer	i.t.	Sun *et al.* 2020
	*Chl.*	Colorectal cancer	i.v.	Wang *et al.* 2021a
	CeCyan	Breast cancer	i.v.	Huo *et al.* 2020b
	Cyan@BPNSs	Breast cancer	i.t.	Qi *et al.* 2021
	UR-Cyan	Breast cancer	i.t.	Qi *et al.* 2021
	*SP*	Head and neck squamous cell carcinoma	/	Saberi *et al.* 2022
	CurNPs-C	Breast cancer	i.v.	Zhang *et al.* 2024b
	*Chl.*	Colon cancer	i.t.	Wang *et al.* 2022
	ALGAE	Breast cancer	i.v.	Zhou *et al.* 2019
	SpiD	Osteosarcoma	i.t.	An *et al.* 2024
	Chlorella AuNRs BSA-Gel	Breast cancer	s.c.	Lee *et al.* 2019
	(Pd@Au)/Fe_3_O_4_@Sp.-DOX microrobots	Esophageal carcinoma renal cell carcinoma	/	Wang *et al.* 2019
	Algae@SiO_2_	Breast cancer	i.v.	Li *et al.* 2020
	CV@CaP	Breast cancer	i.v.	Zhong *et al.* 2021b
	RBCM-Algae	Breast cancer	i.v.	Qiao *et al.* 2020
	MSP hybrid microswimmers	Breast cancer	i.v.	Zhong *et al.* 2020a
	Chl@BP-Fe	Melanoma	i.t.	Ou *et al.* 2022
	S/HSA/ICG	Breast cancer	i.v.	Liu *et al.* 2020
	Au@E-SP	Breast cancer	s.c.	Qi *et al.* 2024
	CP@ICG	Cervicalcancer	i.v.	Zhang *et al.* 2024c
Sono-based	MChl-CQ-HP-NP	Melanoma	i.v.	Gao *et al.* 2023
	*Me*	Breast cancer, Colon cancer	i.v.	Yang *et al.* 2024b
Starvation-based	CP@ICG	Cervicalcancer	i.v.	Zhang *et al.* 2024c
	ACG gel	Breast cancer	i.t.	Zhang *et al.* 2024a
Note: peros (p.o.); intravenous (i.v.); intraperitoneal (i.p.); intratumoral (i.t.); subcutaneous (s.c.)				

### Anti-cancer effect of algae

**Figure 2:**
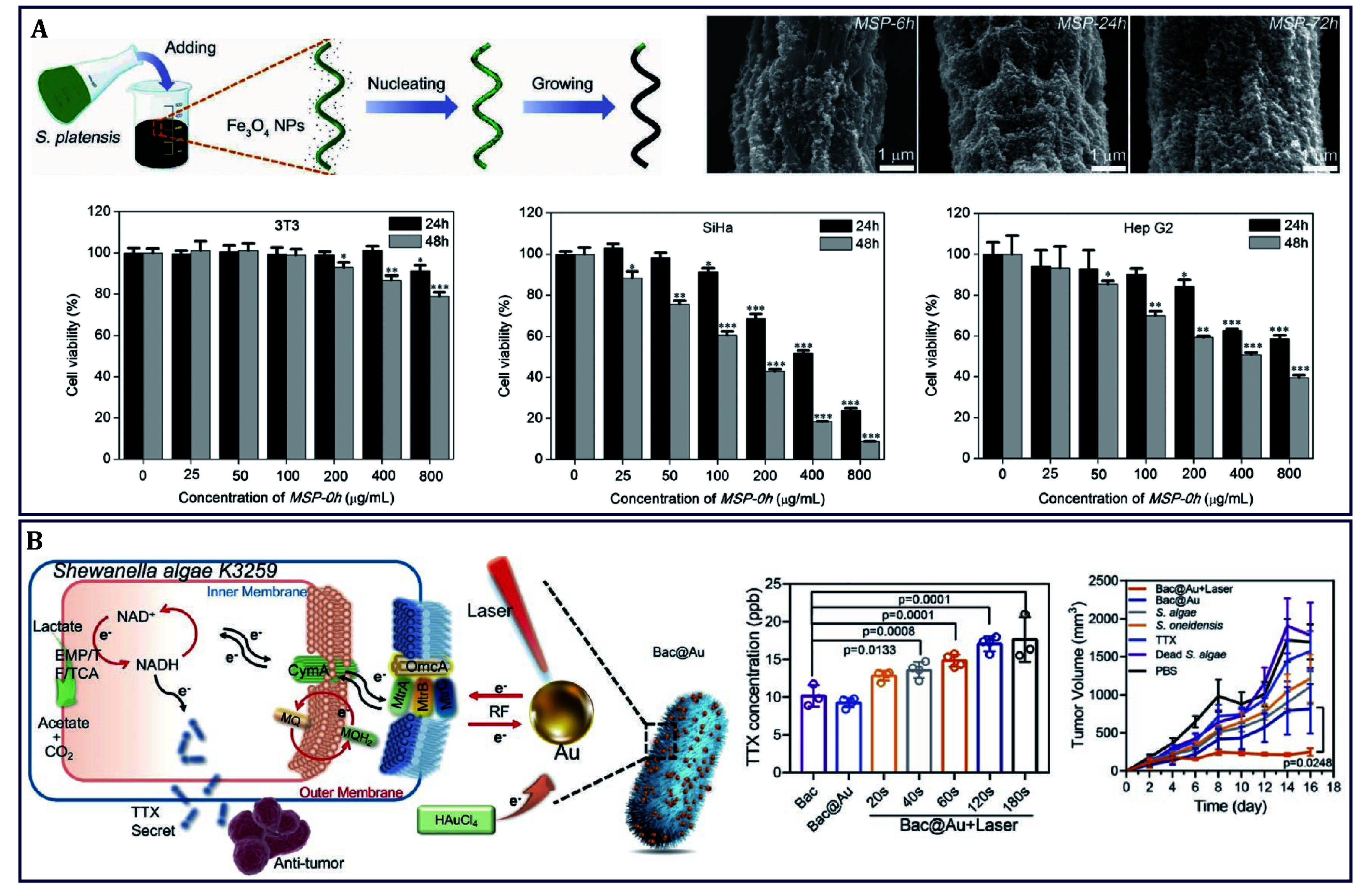
The anti-cancer effects of algae themselves. **A** *SP* delivering Fe_3_O_4_ NPs to act as biohybrid magnetic robot for the selective killing of cancer cells (Yan *et al.* 2017). **B** *S. algae* mediated the *in situ* production of TTX for colon cancer therapy (Wang *et al.* 2021b)

As mentioned above, various algae extracts such as pigments, proteins, saccharides, and nucleic acids, as well as some anti-oxidants, have long been reported to play different roles against cancer. Recently, the potential of algae themselves in cancer therapy has been reported, with different functions inducing mitochondrial-mediated apoptosis, producing anti-cancer immunotoxin and TTX, and selectively targeting cancer cells. For example, a biohybrid magnetic robot that was made of superparamagnetic Fe_3_O_4_ NPs and *SP* with magnetotaxis and spontaneous fluorescence abilities was designed, forming magnetized *SP* (*MSP*) to achieve the selective killing of cancer cells (Fig. 2A) (Yan *et al.* 2017). It was found that *MSP* caused negligible cytotoxicity to 3T3 cells, while the cytotoxicity turned out to be significant in both SiHa and HepG2 cell lines, respectively. To account for the possible selective cytotoxicity, the smaller segments that degraded by *MSP* were found on the surfaces of individual cells and contacted with the cells, which significantly collapsed the cytoskeleton network of cancer cells to induce apoptosis. Furthermore, the released phycocyanin, or particularly C-phycocyanin (C-PC) during degradation was considered the main reason for selective cytotoxicity because it has been evidenced by arresting the cell cycle of cancer cells and further inducing apoptosis, although exhaustive experiments are needed to elaborate this.

In addition to the natural anti-cancer metabolites, engineered algae are promising bioreactors for the *in situ* synthesis of anti-tumor pharmaceuticals within tumors. This approach features the advantages of preventing drug leakage and degradation during delivery. For instance, the neurotoxin TTX could selectively bind and block sodium channels that are formed by transmembrane glycoproteins, which mediate apoptosis by augmenting the oxidative stress level of cancer cells (El-Dayem *et al.* 2013). Nevertheless, the extraction of secondary metabolites from microbial fermentation, such as *S. algae*, resulted in the production of only small quantities of TTX and the consumption of energy. As an energy form, electrons are capable of initiating intracellular metabolic reactions, thereby enhancing the metabolic processes of organisms. Inspired by this, photoelectronic Au NPs that functionalized in transferring optical energy to electrons provided extra exogenous electrons for *Shewanella algae* K3259, a kind of *S. algae*, and then optically controlled Bac@Au system was constructed for *in situ* synthesis of TTX in large quantities against colon cancer (Fig. 2B) (Wang *et al.* 2021b). Under light irradiation, photogenerated electrons produced by Au NPs could accumulate on the surface of bacteria, enter into the cell, promote intracellular metabolism, and increase TTX production, thus mediating cancer therapy. Likewise, *C. reinhardtii*, a eukaryotic green algae, has been reported that immunotoxins containing ribosomal inactivating proteins could be *in situ* produced to reduce the viability of human cancer cells (Tran *et al.* 2013). Not limited to the above-mentioned algae, *Chl.*-based treatment was also reported to cause a significant reduction in tumor volume in a mouse xenograft tumor model via apoptosis (Lin *et al.* 2017). Therefore, no matter whether the anti-cancer substances come from the metabolites or *in situ* synthesis in algae, these novel strategies based on algae themselves would broaden conventional therapy boundaries.

Algae exert anti-cancer ability by inducing mitochondria-mediated apoptosis, producing anti-cancer immunotoxins and TTX, as well as generating selective cytotoxicity in cancer cells, which potentially opens the door to a range of innovative therapies. However, great progress is still needed to unravel the full range of mechanisms behind these phenomena and thus make medical applications more possible.

### Chemo-based cancer therapy

**Figure 3:**
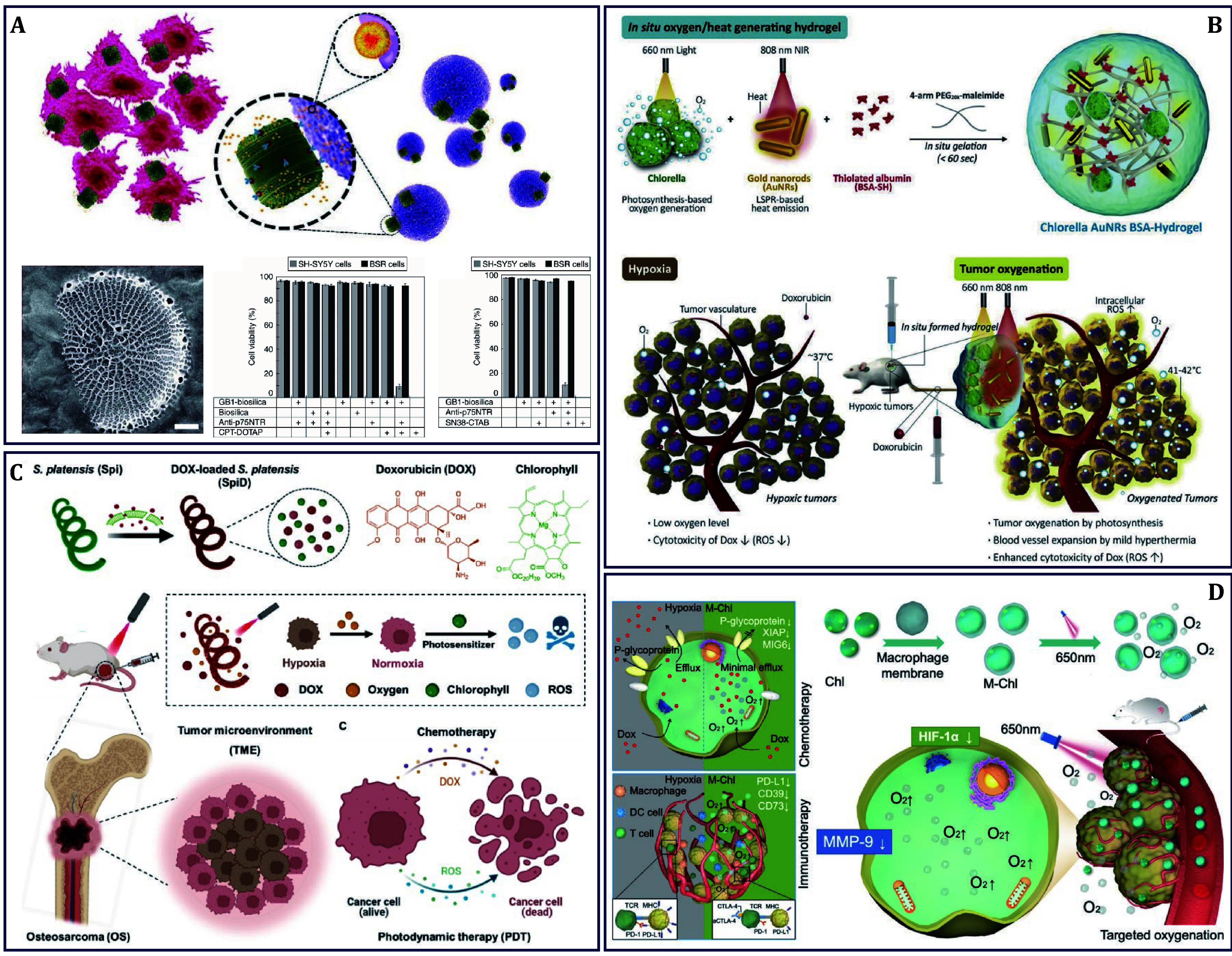
Algae as drug delivery system for chemo-based cancer therapy. **A** *Thalassiosira pseudonana* delivering CPT for chemotherapy of neuroblastoma (Delalat *et al.* 2015). **B** *Chl.* delivering DOX for PTT enhanced chemotherapy of breast cancer (Lee *et al.* 2019). **C** *SP* delivering DOX for combined chemotherapy and PDT of osteosarcoma (An *et al.* 2024). **D** *Chl.* delivering DOX and anti-CTLA-4 for improved chemotherapy and immunotherapy of melanoma (Gao *et al.* 2022)

In clinical practice, chemotherapy is still one of the most extensive and effective means for cancer management, though making patients suffer from serious systemic toxicity. Improving the tumor targeting and enrichment of chemotherapeutic agents to lower the side effects on healthy cells is one of the key goals of chemotherapy. Thanks to the unique morphological structure and surface properties with good biocompatibility and biosafety, algae has become a kind of attractive DDS for optimizing chemotherapy. For example, diatom *Thalassiosira pseudonana* was genetically engineered to display an IgG-binding domain of protein G on the surface of diatom-derived nanoporous biosilica, enabling the attachment of cell-targeting antibodies. Then biosilica as a natural mesoporous silica material was used to deliver chemotherapeutic drugs of CPT and SN38 to target neuroblastoma cells (Fig. 3A) (Delalat *et al.* 2015). B-lymphoma and neuroblastoma cells were selectively destroyed by biosilica exhibiting particular antibodies sorbed with drug-loaded liposome, meaning that genetically engineered biosilica frustules may be used as versatile ‘backpacks’ for the targeted delivery of poorly water-soluble anti-cancer drugs to tumor sites, therefore paving the way for a novel DDS by using a biotechnologically tailored, renewable material.

Besides DDS, expanding the blood vessels within the tumors is another strategy to promote drug entry. Hyperthermia can improve the oxygenation of hypoxic tumors to increase the O_2_ level, therefore, it is considered an effective way to overcome chemotherapeutic resistance. For example, an O_2_-producing and mild febrile hydrogel system of *Chl.,* gold nanorods (Au NRs), and Bovine serum albumin (BSA)-gel, was successfully developed to deliver DOX for photothermal therapy (PTT) enhanced chemotherapy of breast cancer (Fig. 3B) (Lee *et al.* 2019). In this design, the incorporated *Chl*. in the system was able to produce high levels of O_2_ through photosynthesis under 660-nm light irradiation, increasing oxyhemoglobin levels in hypoxic tumors. In addition, 808-nm laser-irradiated Au NRs precisely increased the surrounding temperature to 41–42°C, playing a key role in dilating tumor vessels to overcome chemoresistance. As another type of phototherapy, PDT requires laser excitation to produce ROS instead of heat that can be combined with chemotherapy for a synergistic anti-cancer effect. To this end, a multifunctional DDS of *SP*@DOX was developed for combined chemotherapy and PDT of osteosarcoma (OS) (Fig. 3C) (An *et al.* 2024). With a 650-nm laser irradiation, *SP* generated much O_2_ through photosynthesis and enhanced ROS production through chlorophyll-assisted photosensitization. On this basis, the acidic TME-responsive DOX release cooperated with ROS finally achieved a synergistic killing effect on tumor cells.

In addition to the limited efficiency of O_2_-dependent treatment modalities, the hypoxic TME also contributes to immunosuppression, not just drug resistance. Moreover, the traditional DDS does not work well for O_2_ supply, while the algae-based biological platform showed good sustained release performance (Lim *et al.* 2023). For high-efficiency generating O_2_ and escaping the phagocytosis of immunotherapeutic agents by monocyte phagocytic system (MPS), an algae-based biological platform is by far the better option. As this example showed that macrophage membrane-coated *Chl.* (M-Chl) realized targeted delivery of DOX, antibodies targeting T-lymphocyte-associated protein 4 (CTLA-4), and anti-CTLA-4, for improved chemotherapy and immunotherapy of melanoma (Fig. 3D) (Gao *et al.* 2022). In this structure, *Chl.* camouflaged with macrophage membrane enhanced their tumor accumulation and retention motivated by the inﬂammatory homing effects of macrophage membrane and MPS evading ability, which contributed to sustainable O_2_ production for more than six days, thereby reducing the expression of HIF-1α in the tumor and thereby alleviating oxygen deficiency. After DOX was enriched in the tumor, its sensitivity was significantly improved by inhibiting hypoxia-induced cell efflux, especially the high expression of XIAP. Moreover, the T cell activation agent, an anti-CTLA-4 antibody, was also improved via downregulating hypoxia-mediated immunosuppressive proteins, including CD39, CD73, and programmed death ligand 1 (PD-L1). Therefore, M-Chl has been shown to be an important adjuvant for enhancing the therapeutic effect of anti-cancer drugs in clinics.

Overall, algae is a new platform with great potential for targeted drug delivery. Algae can boost the potency of chemotherapy to selectively kill cancer cells without harming healthy cells. In addition, the high level of O_2_ produced by algae can dilate tumor blood vessels andovercome chemotherapy resistance. Moreover, an algal DDS based on the combination of PDT and chemotherapy can achieve a synergistic effect of DOX and ROS. These results will provide new ideas for developing a green and cost-effective DDS for chemotherapy.

### Radio-based cancer therapy

High-intensity ionizing radiation beams can destroy tumors without depth restriction, making more than half of cancer patients receive RT in clinical settings (Dong *et al.* 2022; Wang *et al.* 2018; Zhong *et al.* 2019). However, the hypoxic TME leads to reduced radiation sensitivity of tumors. Remarkably, the ionizing radiation passing through healthy tissues can also induce severe side effects during RT, especially in the RT of colorectal cancer in the small intestine with a high radiation sensitivity and large organ volume (Billiard *et al.* 2011). Therefore, the simultaneous prevention of radiation-induced intestinal injury is highly desired in RT.

**Figure 4:**
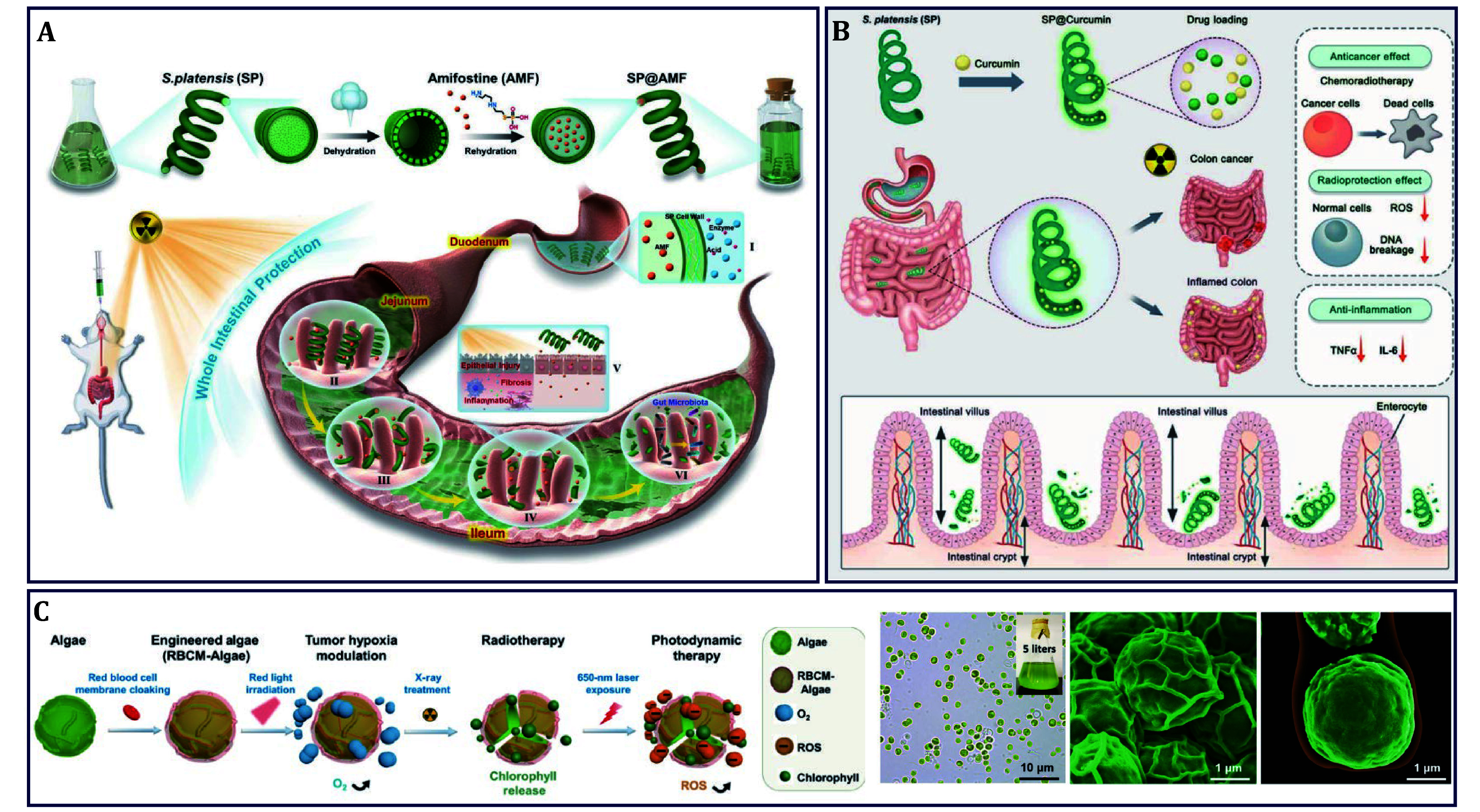
Algae for radio-based cancer therapy. **A** *SP* delivered AMF for RT of orthotopic colorectal tumors with high effective radiation protection against the whole small intestine (Zhang *et al.* 2022). **B** *SP* delivered Curcumin as oral delivery system for combined chemotherapy and RT of colon cancer (Zhong *et al.* 2021c). **C** *C. vulgaris* with the intrinsic photosensitizer were used for combined PDT and RT of breast cancer (Qiao *et al.* 2020)

Currently, the normal tissue radioprotectant of amifostine (AMF) used in clinical has been exposed to some shortcomings such as rapid clearance from blood circulation, conversion into its inactive metabolites by stomach acid, or absorption into circulation in the proximal small intestine, which ultimately result in inadequate accumulation in the small intestine (Augustijns *et al.* 2014; Hensley *et al.* 2009; Obrador *et al.* 2020; Parsons 1977). Algae with O_2_-producing properties via photosynthesis and drug-loading capacities via active surface interactions would provide innovative means for radiosensitization and radiological protection. Previous studies have shown that the helical structure of *SP* made it not only easier to be captured by intestinal villi but also more readily adhered to the intestinal wall, thereby prolonging the retention of the drug in the gut. Based on this, lyophilized *SP* was used as a natural high-efficiency microcarrier to construct an oral delivery system of SP@AMF, with highly effective radiation protection against the whole small intestine during RT of orthotopic colorectal tumors (Fig. 4A) (Zhang *et al.* 2022). In this system, with micron size and progressive degradation *in vivo*, it performed a prolonged and comprehensive intestinal distribution for a selective radioprotective effect on the entire small intestine, while not protecting colon cancer during RT. More interestingly, *SP* promoted the homeostasis of intestinal flora after RT, avoiding the long-term toxicity of AMF. This study provided a new and potentially useful DDS for RT protectants in clinical application. In another work reported by the same group, the payload of AMF was replaced by another FDA-approved anti-cancer drug curcumin, constructed SP@Curcumin as an oral delivery system for the treatment of colon cancer (Fig. 4B) (Zhong *et al.* 2021c). Similarly, *SP* improved the bioavailability and retention time of curcumin in the gastrointestinal tract, realizing combined chemotherapy and RT to inhibit tumor progression. At the same time, SP@Curcumin acted as a radioprotector to remove ROS and reduce ROS-induced DNA damage in healthy tissues. Considering the restrictive effect of hypoxic TME on the effectiveness of RT, a novel *SP*-based whole-cell inorganic-biohybrid system of SP-Au was developed, which could produce O_2_ to alleviate hypoxia under red light irradiation, followed by converting into superoxide anion (·O^2-^) and consuming glutathione (GSH). Combined with the role of Au NCs in intercepting X-rays, enhanced RT was successfully realized (Hua *et al.* 2024). These works provided effective strategies to protect the intestine from radiation damage during RT and would have great potential for clinical application.

Not limited to *SP*, *Chlorella vulgaris* (*C. vulgaris*) with the same function of photosynthesis was also used for hypoxia relief in RT by the same group. Moreover, the chlorophyll in *C. vulgaris* could be used as PSs for PDT to generate ROS under 650-nm light irradiation (Fig. 4C) (Qiao *et al.* 2020). By surface modification with RBCM, the prepared RBCM-Algae achieved reduced uptake by macrophages and systemic clearance. This PDT-RT combined strategy significantly promoted breast cancer cell apoptosis, inducing tumor regression without obvious toxicity. The current studies indicated that photosynthetic algae-based biological systems would provide innovative perspectives for radiation protection and radiation sensitization.

### Immuno-based cancer therapy

Immunotherapy, including immune-checkpoint blockade (ICB) therapy, monoclonal antibody therapy, adoptive cell therapy (TIL), and tumor vaccines has become a revolutionary achievement in metastatic and recurrent tumors (Esmaeilzadeh *et al.* 2023; Yang *et al.* 2022; Zhang and Zhang 2020). However, a limited response rate (< 20%) of patients beneﬁt from immunotherapy was dominated by poor immune cell inﬁltration and immunosuppressive crosstalk in the TME (Pei *et al.* 2024). Therefore, extending the beneﬁts of immunotherapy to more patients is crucial. Many studies have shown that the majority of treatment means such as chemotherapy, can induce the release of tumor-associated antigens (TAAs) from dying tumor cells, followed by triggering immunogenic cell death (ICD) that consequently induces systemic anti-tumor immunity to inhibit distal tumor growth and even prevent tumor reoccurrence (Li *et al.* 2024a). The application of algae in immunotherapy allows them to regulate the hypoxic TME through photosynthesis, and mediate combination treatment strategies, thus triggering robust immune effects after reversing the immunosuppressive TME. Moreover, algae can be considered as a kind of immune adjuvant to promote the infiltration and efficacy of immune cells. These advantages make algae one of the strategies to enhance immunotherapy (Qi *et al.* 2024).

**Figure 5:**
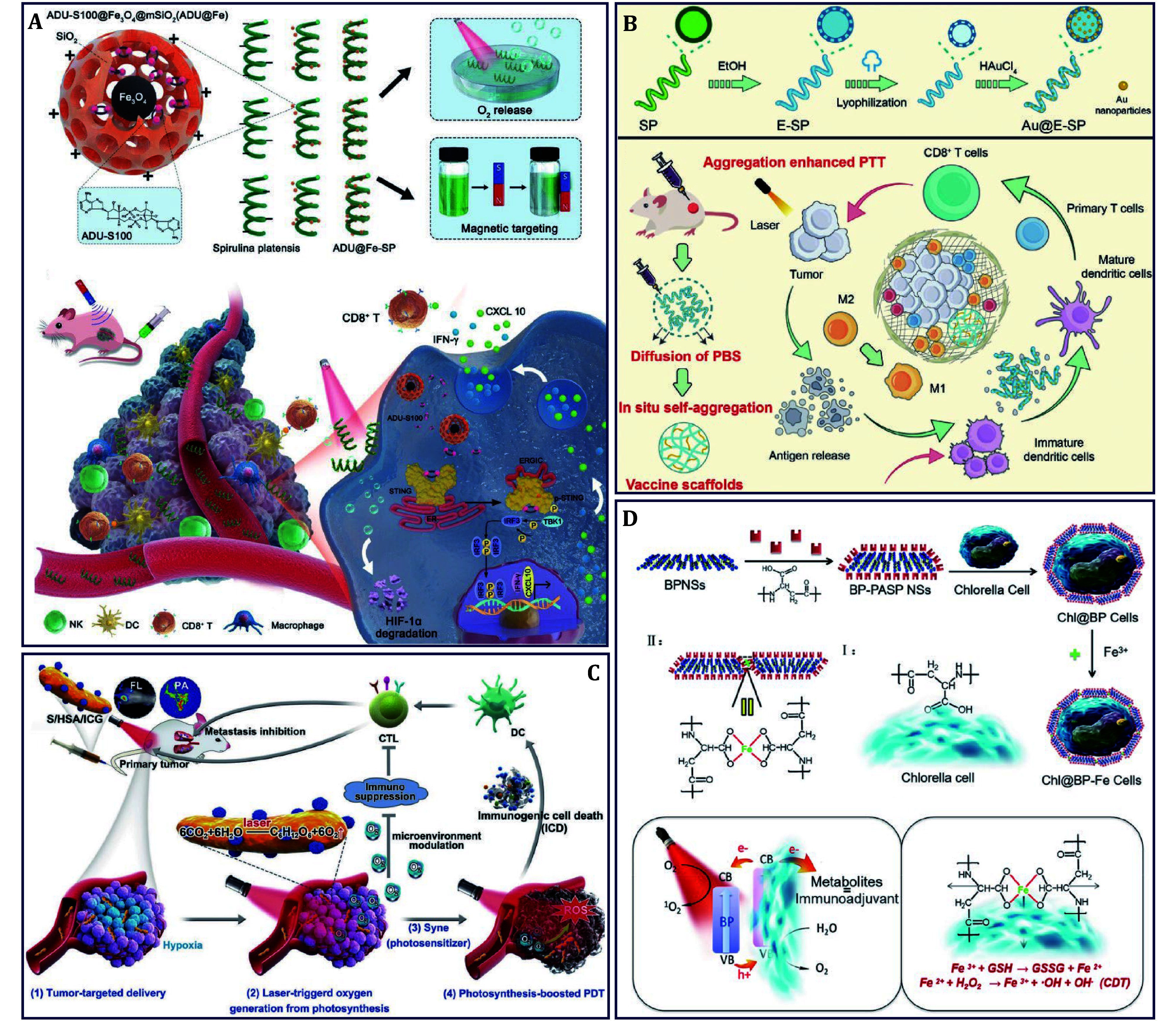
Algae for immuno-based cancer therapy. **A** *SP* delivering Fe_3_O_4_ and ADU for immunotherapy of colon cancer (Zhang *et al.* 2023). **B** *SP* delivering Au NPs for combined PTT and immunotherapy of breast cancer (Qi *et al.* 2024). **C** *Syne* delivering ICG for PDT-immunotherapy of breast cancer (Liu *et al.* 2020). **D** *Chl.* delivering Fe contained BPNSs through “Lego building method” for cascade promoted PDT-CDT-immunotherapy of melanoma (Ou *et al.* 2022)

Recent studies have shown that hypoxia can not only hinder proliferation and differentiation of immune cells but also favor immune evasion and resistance to weaken the innate immune responses (Yang *et al.* 2023b). Although several chemotherapeutic drugs could train the immune system to some extent, the alleviated outcome was observed when exposed to a hypoxic TME. Thus, hypoxia relief is an intervention target for effective immunotherapy. ADU-S100 (ADU) is the agonist of stimulator of interferon gene (STING) that suffers from a short biological half-life, susception to nucleases, and lack of targeting, which means it requires intra-tumoral injection in most situations. To solve these, the biological hybrid system of ADU@FeSP was constructed for enhanced immunotherapy of colon cancer (Fig. 5A) (Zhang *et al.* 2023). Wherein, *SP* acted as an *in situ* oxygen factory for the alleviation of hypoxic TME, restoring stimulator of interferon gene/TANK-binding kinase 1/interferon regulatory factor 3 (STING/TBK1/IRF3) signaling and immune cells activation, while Fe_3_O_4_@mSiO_2_ NPs afforded *SP* with magnetic targeting ability to deliver ADU, which played a collaborative role in remodeling the immunosuppressive TME and provided a promising strategy for boosting anti-tumor immunity.

Tumor vaccines are an active immunotherapy approach by harnessing the immune system of hosts, which will inhibit tumor development and promote long-term immune memory. However, considering the limited immunogenicity of tumor antigens and the immunosuppressive TME, the use of adjuvants and vaccine carriers holds great potential for enhancing immune effects (Bo and Wang 2024). Various stimuli like drugs, radiation, heat, and ROS, can *in situ* form tumor vaccine by presenting a full spectrum of TAAs. In addition, *SP* has been reported with the ability to activate human innate immunity, while its potential as an immune adjuvant scaffold has not been explored. It was found that the adjuvant potential, *in situ* vaccine scaffold of Au@E-SP based on inactivated *SP* (E-SP) and Au NPs could enhance local hyperemia and activate the anti-tumor immune response toward breast cancer (Fig. 5B) (Qi *et al.* 2024). The *SP* ﬁbers could adsorb Au^3+^ ions for *in situ* synthesis of photothermal gold NPs. After subcutaneous injection, Au@E-SP could be self-aggregated; the Au NPs were concentrated and significantly enhanced the local PTT and thus induced the release of TAAs. Moreover, as a natural immune adjuvant, the retained E-SP promoted the reprogramming of macrophages from the M2 into the M1 subtype, modulating the immunosuppressive TME, and improving the efficacy of thermal immunotherapy to a large extent. In addition to PTT-induced ICD, PDT-mediated ICD also generates vaccine-like functions *in situ* and does not need foreign antigens. For example, a natural photosynthetic *Synechococcus 7942* (*Syne*) was used as tumor-targeted photosensitizer delivery for hypoxia alleviated photodynamic-immunotherapy of metastatic triple-negative breast cancer (Fig. 5C) (Liu *et al.* 2020). In detail, the photosensitizer ICG was encapsulated into HSA NPs by intermolecular disulfide crosslinking, forming HSA/ICG NPs that could attach to the surface of *Syne* with amide bonds. The final prepared S/HSA/ICG combined the photosynthetic capability of a moderate immune stimulation effect of *Syne*, as well as the PDT effect of HSA/ICG under 660-nm laser irradiation. Upon intravenous injection, S/HSA/ICG effectively accumulated around the tumor site and eliminated primary tumors; this system even robustly evoked systematic anti-tumor immunity to prevent tumor recurrence and metastasis.

From the perspective of complex TME that brings obstacles to cancer treatment, the hypoxic environment can lead to chemo-, radio-therapy resistance, and T cell depletion. However, by ingenious harnessing, it also provides opportunities to explore new therapeutic strategies, like chemodynamic therapy (CDT) that specifically utilizes the acidic TME with a high concentration of hydrogen peroxide (H_2_O_2_) and appropriate Fenton/Fenton-like agents (Wang *et al.*2020b; Zhao *et al.* 2023; Zhong *et al.* 2021d). Considering the further combination of CDT on top of the PDT-induced immune response achieved by algae, Chl@BP-Fe constructed through the “Lego building method” realized cascade promotion of PDT-CDT-immunotherapy in melanoma (Fig. 5D) (Ou *et al.* 2022). As one of the building blocks, *Chl.* with inherent photosynthesis effectively ameliorated tumor hypoxia for promoted T cell infiltration; it also acted as an immune adjuvant which stimulated the maturation of dendritic cell (DC). Meanwhile, the extracellular BPNSs and intracellular chlorophyll of *Chl.* together constructed a type-II heterojunction, which improved the conversion efficiency of light via “electron-hole separation” for ^1^O_2_ generation and O_2_ evolution, respectively. Additionally, Fe^3+^ ions consuming GSH not only reduced the reductive TME but also turned into Fe^2+^ ions to catalyze the Fenton reaction with H_2_O_2_ for ⋅OH generation that mediates CDT. This system thereby successfully reduced tumor metastasis.

Altogether, these *in situ* aggregated vaccines hold great promise in guaranteeing high potential for clinic applications by taking advantage of natural materials in cancer immunotherapy. The successful implementation of these studies provides a promising immunogenic strategy for the application of algae in tumor immunotherapy basing on relieving hypoxia. At the same time, a photodynamic/chemodynamic/immune synergistic strategy has been proposed to solve the problems of hypoxia inhibition and insufficient immune stimulation, which provides a new idea for tumor immunotherapy.

### Photo-based cancer therapy

Light, used in the field of biomedical application, can be dated back to the 19^th^ century when UV irradiation was initially employed to treat lupus vulgaris, which opened the era of phototherapy. Through decades of endeavors, PTT and PDT have become one of the most popular clinical therapeutic modalities against a variety of diseases such as cancer (Sun *et al.* 2023). PTT relying on photothermal agents achieves the purpose of tumor elimination by thermal effect, while PDT is based on PSs for ROS production to kill cancer cells (Hosseini *et al.* 2022; Shan *et al.* 2024). Both methods have the advantages of high efficacy, good controllability, and minimal invasive damage. However, the traditional photothermal agents and PSs synthesized at harsh conditions such as high temperature and high pressure would make them potentially toxic and slow removal from the body. Algae ensures the biological safety of its clinical application, making it an important candidate to participate in the design of novel photo-responsive agents (Sun *et al.* 2020).

**Figure 6:**
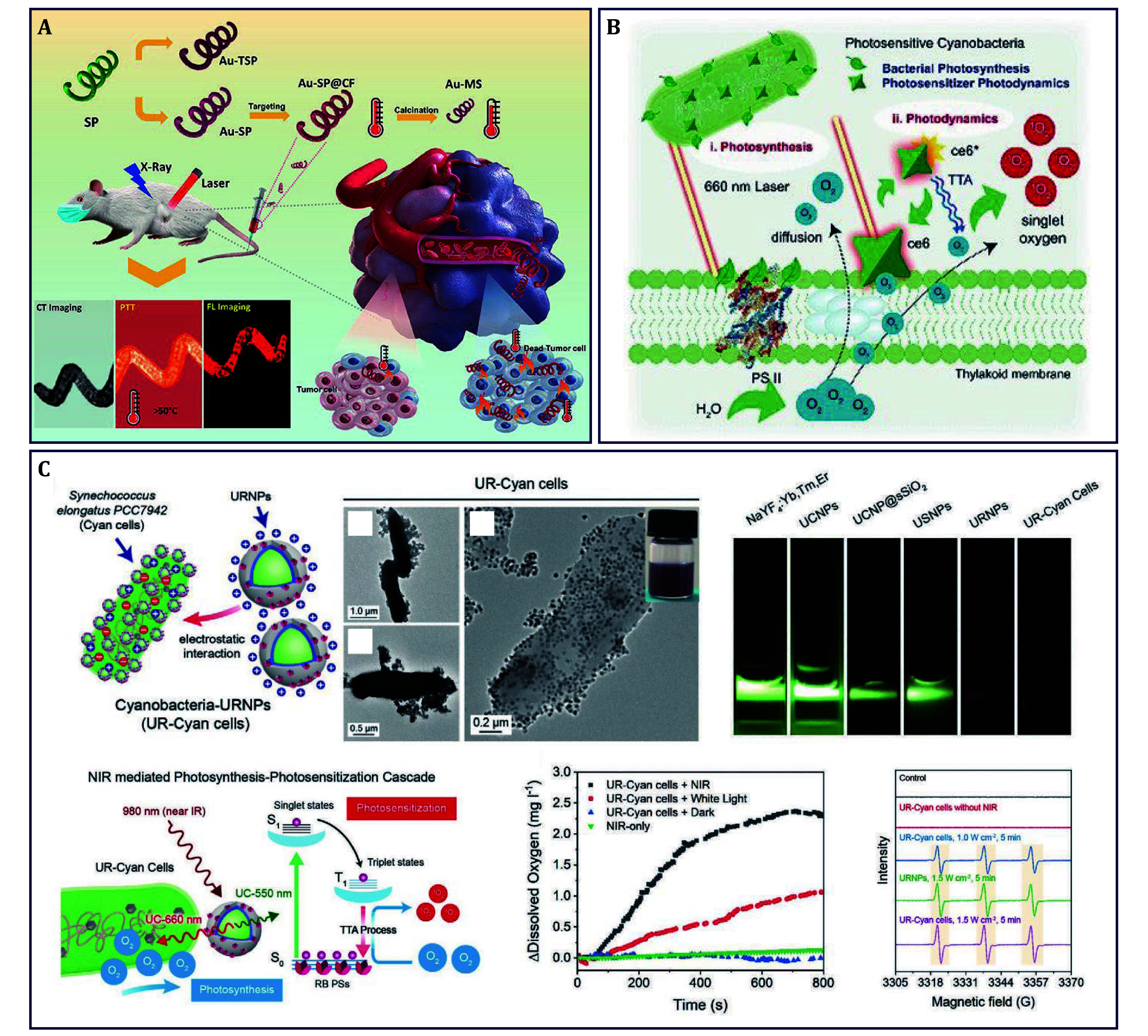
Algae for photo-based cancer therapy. **A** *SP* delivering Au NPs for PTT of breast cancer (Sun *et al.* 2020). **B** *Cyano* delivering Ce6 for PDT of breast cancer (Huo *et al.* 2020b). **C** *Cyano* delivering RB-loaded UCNPs for PDT of breast cancer (Huo *et al.* 2021)

Au NPs have been reported as a biocompatible *in vivo* photothermal agent, therefore, *SP* was utilized as the synthesis template to construct the helical architecture of quasi-spherical Au NPs (Au-SP) and further engineered with targeting moiety of folic acid. Therefore, a safe, biodegradable, and tumor-targeted biohybrid of Au-SP@CF was prepared for PTT of triple-negative breast cancer (Fig. 6A) (Sun *et al.* 2020). Of particular note, high tumor inhibition effects were obtained by the excellent photothermal performance of Au-SP@CF, indicating the key role of the helical architecture of Au NPs in achieving a high photothermal effect. In short, this strategy with well-controllable immobilization of Au NPs, appropriate biodegradability, high tumor suppression, as well as low tumor metastasis effects pave a new way for the design and manufacture of advanced photothermal agents.

At present, PDT is evidenced to be effective against superficial tumors with a satisfactory prognosis, especially for type-II PDT, sustained tumor oxygenation via photosynthetic algae is of great essential. For example, *Cyan* was modified with Ce6 to construct ceCyan for PDT of breast cancer (Fig. 6B) (Huo *et al.* 2020b). Under 660-nm laser irradiation, this photosensitive ceCyan remarkably supplied O_2_ and then quickly transformed O_2_ to ^1^O_2_ for PDT. Next year, this group replaced Ce6 with a photosensitizer of BPNSs to mediate 660-nm laser-excited PDT, extending the scope of PDT by hybridizing algae with inorganic nanophotosensitizer (Qi *et al.* 2021). However, O_2_ supply alone does not fully exert efficacy; it is needed to enrich the O_2_ around the PSs during PDT (Herlambang *et al.* 2011; Sortino *et al.* 2006; Zhu *et al.* 2016). Therefore, a novel light-controlled sustainable PDT based on *Chl.* was proposed, which enriched the O_2_ around the ICG by perfluorocarbons (PFCs) to maintain the O_2_ level. After the cessation of light exposure, *Chl.* further acted as an adjuvant to promote DC activation and promoted the anti-tumor immune response (Wang *et al.* 2021a). In fact, the extremely limited laser penetration depth of visible light is one of the most critical factors against PDT for deep-seated tumors. Alternatively, near-infrared (NIR) light with higher tissue penetration ability has been utilized to excite PSs for deep PDT via the process of light conversion. UCNPs are a kind of popular energy converters that can specifically absorb two or more low-energy photons to emit a high-energy photon. By optimizing the composition and proportion of the rare-earth (RE) ions, UCNPs can be designed to turn NIR light and emit UV/vis light with adjustable wavelength. Therefore, this group further developed a NIR-driven PDT platform of UR-Cyan, through RB-loaded UCNPs wrapping on the surface of *Cyan*, for PDT of breast cancer (Fig. 6C) (Huo *et al.* 2021). Basically, the NaYF_4_:Yb^3+^,Tm^3+^,Er^3+^@NaYF_4_:Yb^3+^ core-shell UCNPs were able to transfer 980-nm laser to 456-, 481-, 520-, 540-, 654-, and 651-nm light, a wide wavelength range covering the absorption of RB (500–580 nm). This fluorescence resonance energy transfer (FRET)-based photocatalytic process allowed to release of O_2_ by *Cyan* and produce ^1^O_2_ by RB, thus realizing robust tumor regression.

The algae-based multifunctional integrated PDT microsystem expands the application of microorganisms in the biomedical field, especially enhancing the role of PDT in the treatment of cancer. In addition, *Chl.* has the dual effects of O_2_ production and immunity enhancement, which may have a positive effect on improving the systemic anti-cancer immune response after PDT. In conclusion, this algae-based biological system provides a new perspective for the development of cancer photo-therapies with active chemotaxis and efficient microenvironment regulation.

### Sono-based cancer therapy

Sonodynamic therapy (SDT) can control tumor growth while limiting toxicity in a non-invasive manner (Yang *et al.* 2024a; Yang *et al.* 2022). It uses low-frequency and low-intensity ultrasound (US) to trigger sonosensitizers and then kills tumor cells with ROS (Cen *et al.* 2021; Yang *et al.* 2024c). However, due to the lack of sonosensitizers and the hypoxic TME, the outcomes of SDT on tumors are still limited (Loke *et al.* 2023; Wang *et al.* 2020a; Yang *et al.* 2023c). In recent years, the inherent O_2_ production capacity and US-responsive structure or components have made algae a candidate for enhanced SDT.

**Figure 7:**
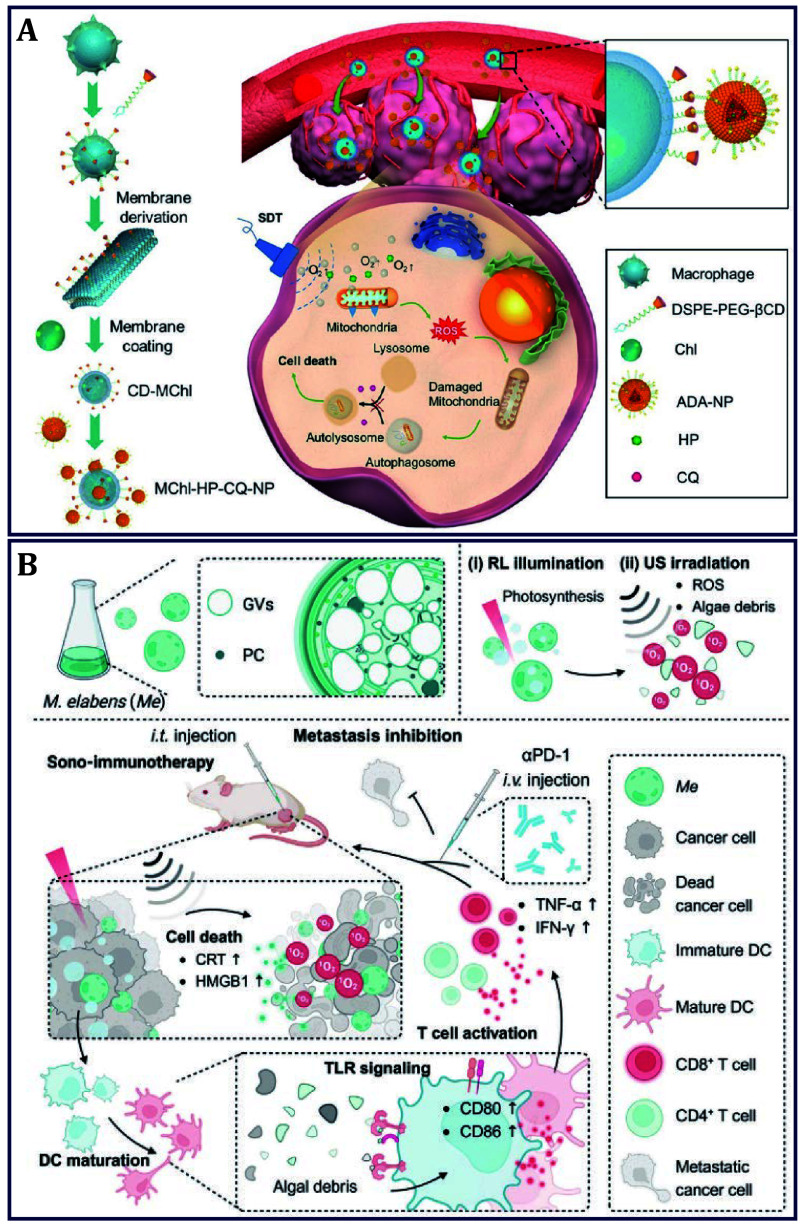
Algae for sono-based cancer therapy. **A** *Chl.* delivering HP and CQ for combing SDT and autophagy inhibition of melanoma (Gao *et al.* 2023). **B** *Me* as natural sonosensitizer for eﬃcient SDT-ICB for colon and breast cancer therapy (Yang *et al.* 2024b)

Autophagy, a protective pathway of tumor cells against oxidative stress, can facilitate cellular survival by attenuating apoptotic cell death, thus compromising the efficacy of SDT. To realize augmented SDT via inhibiting autophagy, MChl-CQ-HP-NP was constructed as the supramolecular conjugate for the treatment of melanoma (Fig. 7A) (Gao *et al.* 2023). In this structure, *Chl.* was first coated with *β*-cyclodextrin (*β*-CD) modified macrophage membrane to bestow CD-MChl with lowered inflammatory response, reduced elimination, and increased accumulation. Then, the sonsosensitizer hematoporphyrin (HP) and autophagy inhibitor chloroquine phosphate (CQ) were carried by adamantane (ADA) modified liposome (ADA-NP), and further conjugated to MChl via host-guest interactions between *β*-CD and ADA. The MChl hitchhiking delivery not only facilitated tumor homing of dual drugs but also alleviated hypoxia, which resulted in resistance to rechallenging inoculation of melanoma cells by strong anti-tumor immune memory of the treated mice. In addition to the algae as DDS for sonosensitizer delivery, phycocyanobilin (PC) for photosynthesis within the cells has already been demonstrated to be a photosensitizer. Moreover, the intracellular gas-ﬁlled gas vesicles (GVs) for ﬂoating are natural cavitation nuclei that have the potential to improve sonosensitization eﬃciency. By fully exploiting these advantages, our group expanded these underestimated microalgae to the application of SDT. Here, *Microcystis elabens* (*Me*), was chosen to develop a simple yet eﬃcient sono-immunotherapy for combating colon and breast cancer (Fig. 7B) (Yang *et al.* 2024b). Routinely, *Me* generated O_2_ upon exposure to red light to modulate hypoxia. What was innovative was that GVs acted as cavitation nuclei, which facilitated US-triggered ROS generation by the natural sonosensitizer of PC. Moreover, algal debris could serve as vaccine-like functions to further activate the host immune system against cancer, which signiﬁcantly promoted DC maturation via Toll-like receptor (TLR) signaling pathways, augmented differentiation of CD8^+^ and CD4^+^ T cells, and thus largely inhibited tumor growth and extended the survival time of mice. Considering the possibility of immune tolerance, the *Me*-based sono-immune strategy was further combined with ICB therapy to reverse immunosuppression and expand its applications. Satisfactory outcomes were obtained with robust abscopal effects and encouraging immune memory effects. This work confirmed algae with outstanding sonosensitizer potential to be clinically transformed.

At present, SDT still faces challenges due to limited sonosensitizers and hypoxic TME. The O_2_-producing and US-responsive properties of algae provide a solution for enhanced SDT. At the same time, combined with other therapeutic methods can further improve the curative effect, algae has great clinical potential in SDT.

### Starvation-based therapy

Starvation therapy emerges as an effective method for suppressing tumor growth through depriving essential nutrients/oxygen supply via angiogenesis inhibiting agents (AIAs), vascular disrupting agents (VDAs), and transarterial chemoembolization (TACE) mediated blood occlusion. Unfortunately, various limitations including poor bioavailability, rapid metabolism, elevated tumor hypoxia, acute coronary syndromes, abnormal ventricular conduction, induced drug resistance, and increased tumor metastasis risk, finally impede their further applications in the clinic (Yu *et al.* 2019). The advances show that algae play an active role in mediating tumor metabolism, for example, consuming glucose in tumors while producing O_2_ through photosynthesis, broadening the application prospects of starvation therapy for cancer.

**Figure 8:**
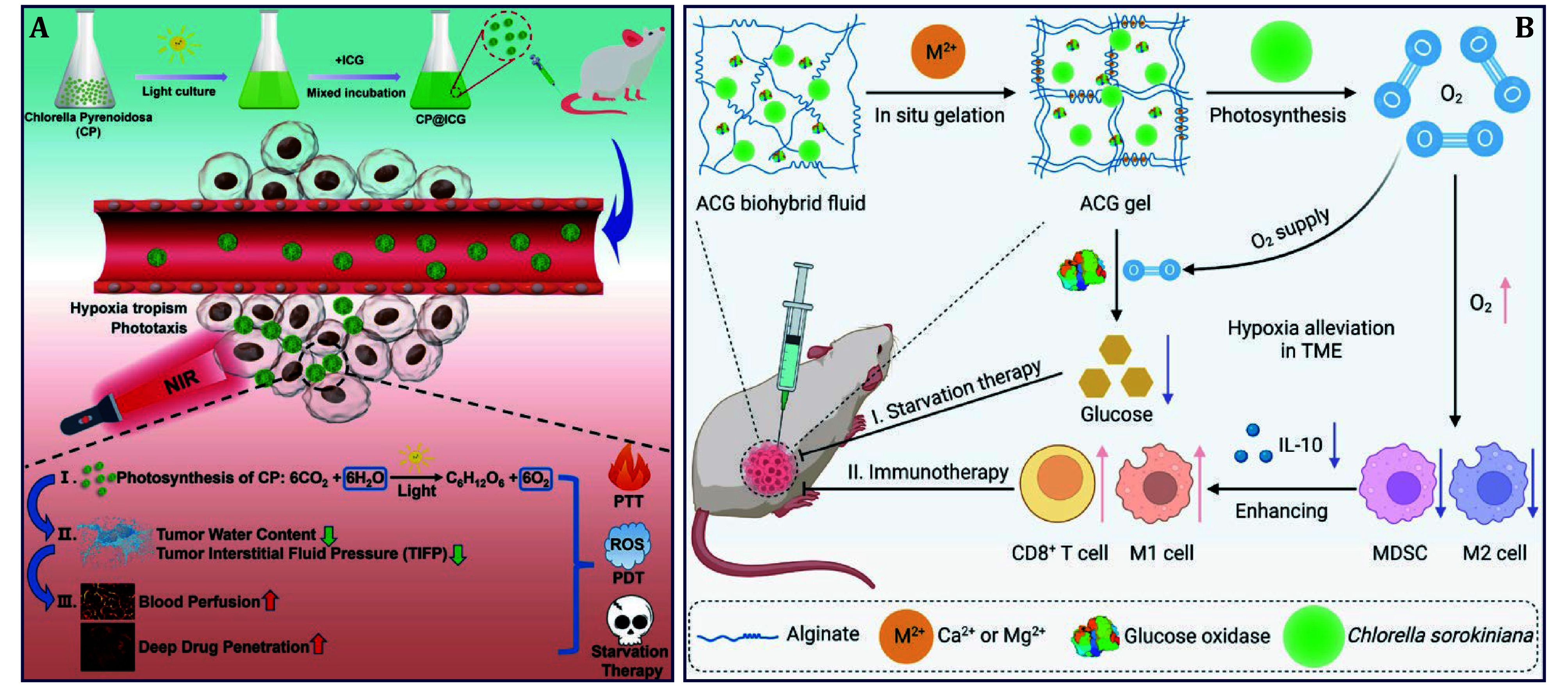
Algae for starvation-based cancer therapy. **A** *CP* delivering ICG for PTT/PDT/starvation therapy of breast cancer (Zhang *et al.* 2024c). **B***C. soro* delivering GOx for starvation therapy and immunotherapy of breast cancer (Zhang *et al.* 2024a)

*Chlorella Pyrenoidosa* (*CP*) possessing hypoxia tropism, phototropism, and photosynthetic water-splitting capabilities allows it to actively target tumor sites for starvation therapy. For example, ICG was loaded onto *CP* forming CP@ICG composite for PTT/PDT/starvation therapy of breast cancer (Fig. 8A) (Zhang *et al.* 2024c). Upon exposure to 660-nm NIR light, *CP* decomposed excess water within the tumor and produced O_2_, thereby alleviating tumor hypoxia and providing an O_2_ source for augmented PDT. Under 808-nm light irradiation, ICG realized excellent photothermal and photodynamic therapeutic effects. Concurrently, *CP* proliferated locally within the tumor, which continuously consumed glucose for achieving starvation therapy. This research presented a new paradigm for the application of algae in oncotherapy.

Besides photosynthesis via splitting water, glucose oxidase (GOx) can effectively catalyze the oxidization of glucose into gluconic acid and H_2_O_2_ in the presence of O_2_, which renders GOx a great application prospect in starvation therapy (Fu *et al.* 2019). Next, another study integrated algae and GOx within a hydrogel for a comprehensive breast cancer attack by combining starvation therapy and immunotherapy (Fig. 8B) (Zhang *et al.* 2024a). In detail, the ACG gel was *in situ* formed after intratumoral injection of the alginate/*Chlorella* *sorokiniana* (*C. soro*)/GOx biohybrid fluid via cross-linking between Ca^2+^ or Mg^2+^ ions within tumors. Under 660-nm laser irradiation, abundant O_2_ was generated from *C. soro*-mediated photosynthesis, which not only reoxygenated hypoxia tumor tissues, but significantly enhanced tumor starvation therapy by supplying O_2_ for the glucose consumption catalyzed by GOx. Moreover, hypoxia alleviation reshaped immuopermissive TME, resulting in reducing myeloid-derived suppressor cells (MDSCs) and M2 phenotype macrophages while increasing M1 phenotype macrophages and CD8^+^ T cells; this treatment also improved the effectiveness of PD-1 blockade. Overall, this study highlighted the possibility of algae for enhancing hypoxia-constrained tumor starvation therapy and immunotherapy.

Cancer starvation therapy inhibits tumor growth by blocking nutrient supply, but its clinical application is still limited due to low bioavailability and increased risk of metastasis. Algae have a two-pronged approach from two perspectives: consuming glucose and producing oxygen in tumors, which fuel the progress of starvation therapy.

## SUMMARY AND OUTLOOK

In recent years, algae have led to a growing interest in cancer management owing to their unique anti-tumor properties. Under the auspices of tailored functional NMs, these superior properties of algae have ushered novel prospects in both traditional means and newly developed strategies for broad-spectrum cancer therapy. In this review, we aim to provide a systematic summary of algae-based advanced platforms in the realm of oncotherapy. Although being successful in achieving better anti-cancer effects that indicate a bright research prospect, an array of existing obstacles remain to be surmounted.

(1) Manufacturability and controllability (Jia *et al.* 2024). For physical or chemical modiﬁcation of algae, current studies mainly focus on the modiﬁcation of cell surfaces. We anticipate the intracellular modiﬁcations of algae will be a research point in the future. In addition, the nature of NMs may affect the growth, metabolism, and activity of algae. Thus, achieving precise *in vitro* control is a significant challenge that needs to be addressed. Furthermore, other stimuli-responsive NMs can be combined with algae for innovative treatment strategies such as electro-, thermo-, and radiodynamic-based approaches.

(2) The complexity of the interactions *in vivo* (Chen *et al.* 2021). Different from the algae extract in the ingredients are relatively clear, the intricate interplay between algae and microbiota introduces complexity that could potentially affect the consistency and predictability of treatment outcomes. It is imperative to delve deeper into these intricate dynamics in the trajectory of future research endeavors that probably contribute to reﬁning algae-based treatment strategies.

(3) The anti-cancer mechanisms are limited (Xin *et al.* 2023). Despite lots of efforts made, the effects of algae on immunomodulation are poorly studied, limiting the understanding of the anti-cancer effects of algae. More researches are needed to uncover their activity in immunotherapy.

(4) Safety (Zhong *et al.* 2021a). The *in vivo* degradation/recycling pathways post the exertion of algae remain unclear. Researchers need to comprehensively evaluate the potential toxicity, allergy, and impact on the host physiological functions of engineered algae. In addition, avoiding the potential pathogenicity and spread of algae is also key to ensuring safety. More clinical studies are still necessary to confirm the long-term toxicity and immunogenicity of various algae before their application in clinical treatment.

(5) Others. The limited survival of algae and drug loading capacity, the lack of large-scale production, as well as the pending clinic trial, all restrict the progress of algae in cancer therapy implementation. Establishing standardized therapeutic regimens, such as determining the precise dosage of administration, defining the optimal duration, ascertaining the accumulation of algae at the tumor site, and identifying monitoring indicators, are of paramount importance to ensure the successful application of algae-based therapy.

To make substantial progress and realize sustainable development in the field of cancer control, interdisciplinary research initiatives must be broadened to examine and construct engineered algae with the desired functionalities. Once achieved, the algae-based cancer therapies will be invaluable clinical tools for cancer prevention and detection, ultimately offering unprecedented avenues for advanced cancer therapy.

## Conflict of interest

Tian Qiu, Xingrun Li, Hui Sun, Simeng Zhang, Yan An, Jianxiang Li and Xiaoyan Zhong declare that they have no conflict of interest.
